# Comment on Rotaru et al. Lean MASLD and IBD: Exploring the Intersection of Metabolic Dysfunction and the Gut–Liver Axis. *Life* 2025, *15*, 288

**DOI:** 10.3390/life15081210

**Published:** 2025-07-30

**Authors:** Giuseppe Guido Maria Scarlata, Ludovico Abenavoli

**Affiliations:** Department of Health Sciences, University “Magna Græcia”, 88100 Catanzaro, Italy; l.abenavoli@unicz.it

We read with great interest the recent article by Rotaru et al. on the crucial role of the gut–liver axis in the lean metabolic dysfunction-associated steatotic liver disease (MASLD) and inflammatory bowel disease (IBD) shared pathogenesis [1]. The recent redefinition of nonalcoholic fatty liver disease (NAFLD) to MASLD represents a paradigm shift in hepatology. This new nomenclature focuses on the presence of hepatic steatosis in the context of cardiometabolic risk factors, such as overweight/obesity, insulin resistance, and hypertension, without relying on the exclusion of significant alcohol intake or other liver diseases [2]. Importantly, it acknowledges the heterogeneity of patients previously classified under NAFLD, including those who are lean but exhibit metabolic disturbances [3]. A critical component in the development and progression of MASLD is the gut–liver axis. The bidirectional communication between the gut and the liver is mediated through the portal circulation, whereby gut-derived microbial products, dietary metabolites, and inflammatory mediators directly influence hepatic physiology [4]. In IBD, gut dysbiosis and mucosal barrier disruption lead to increased intestinal permeability and bacterial translocation. These processes trigger hepatic inflammation via activation of pattern recognition receptors, such as toll-like receptors, and stimulate hepatic stellate cells, contributing to fibrogenesis [5]. Furthermore, altered bile acid signaling and disequilibrium of gut microbiota in IBD can disturb lipid metabolism and insulin sensitivity, thereby exacerbating hepatic steatosis and metabolic dysfunction [6]. The MASLD definition is particularly relevant in highlighting disease phenotypes beyond the classical overweight/obese individual, notably in lean patients who may present with metabolic derangements. By focusing on metabolic criteria rather than body mass index alone, the MASLD framework incorporates a broader spectrum of patients, including those with IBD, who may be lean but remain at risk due to chronic inflammation [7,8]. This inclusive approach enhances the clinical identification of at-risk individuals and facilitates research into the unique mechanisms driving MASLD in lean populations and those with chronic inflammatory comorbidities [9]. However, a recent study conducted by our research group in an IBD cohort compared the two different nomenclatures, NAFLD and MASLD, from anthropometric, clinical, and laboratory perspectives. In this context, the IBD-MASLD population was significantly more overweight and exhibited significantly higher frequencies of other cardiometabolic parameters, such as type 2 diabetes and hypertension, compared to the IBD-NAFLD group [10]. Therefore, although the new MASLD definition includes a lean phenotype, it is important to consider that in a high-risk population such as patients with IBD, this definition may largely encompass overweight or obese individuals. Further longitudinal studies in larger patient cohorts are needed to better elucidate the crucial role of the gut–liver axis in IBD-MASLD pathogenesis. Notably, such investigations should also include genomic and expression analyses, which could provide prognostic signatures to stratify patients and predict outcomes.

## Abbreviations

The following abbreviations are used in this manuscript:

IBD	Inflammatory Bowel Disease
NAFLD	Nonalcoholic Fatty Liver Disease
MASLD	Metabolic Dysfunction-associated Steatotic Liver Disease
